# Hispanic physicians' tobacco intervention practices: a cross-sectional survey study

**DOI:** 10.1186/1471-2458-5-120

**Published:** 2005-11-14

**Authors:** Francisco G Soto Mas, Richard L Papenfuss, Holly E Jacobson, Chiehwen Ed Hsu, Ximena Urrutia-Rojas, William M Kane

**Affiliations:** 1 Department of Social and Behavioral Sciences, School of Public Health, University of North Texas Health Science Center, Fort Worth, Texas, USA; 2 Department of Health Promotion, University of Nevada Las Vegas, Las Vegas, Nevada, USA; 3 Department of Kinesiology, Health Promotion & Recreation, University of North Texas, Denton, Texas, USA; 4 Department of Public and Community Health, University of Maryland College Park, College Park, Maryland, USA; 5 Department of Physical Performance and Development, Health Education Program, University of New Mexico, Albuquerque, New Mexico, USA

## Abstract

**Background:**

U.S. Hispanic physicians constitute a considerable professional collective, and they may be most suited to attend to the health education needs of the growing U.S. Hispanic population. These educational needs include tobacco use prevention and smoking cessation. However, there is a lack of information on Hispanic physicians' tobacco intervention practices, their level of awareness and use of cessation protocols, and the type of programs that would best address their tobacco training needs. The purpose of this study was to assess the tobacco intervention practices and training needs of Hispanic physicians.

**Methods:**

Data was collected through a validated survey instrument among a cross-sectional sample of self-reported Hispanic physicians. Data analyses included frequencies, descriptive statistics, and factorial analyses of variance.

**Results:**

The response rate was 55.5%. The majority of respondents (73.3%) were middle-age males. Less than half of respondents routinely performed the most basic intervention: asking patients about smoking status (44.4%) and advising smoking patients to quit (42.2%). Twenty-five percent assisted smoking patients by talking to them about the health risks of smoking, providing education materials or referring them to cessation programs. Only 4.4% routinely arranged follow-up visits or phone calls for smoking patients. The majority of respondents (64.4%) indicated that they prescribe cessation treatments to less than 20% of smoking patients. A few (4.4%) routinely used behavioral change techniques or programs. A minority (15.6%) indicated that they routinely ask their patients about exposure to tobacco smoke, and 6.7% assisted patients exposed to secondhand smoke in understanding the health risks associated with environmental tobacco smoke (ETS). The most frequently encountered barriers preventing respondents from intervening with patients who smoke included: time, lack of training, lack of receptivity by patients, and lack of reimbursement by third party payers. There was no significant main effect of type of physician, nor was there an interaction effect (gender by type of physician), on tobacco-related practices.

**Conclusion:**

The results indicate that Hispanic physicians, similarly to U.S. physicians in general, do not meet the level of intervention recommended by health care agencies. The results presented will assist in the development of tobacco training initiatives for Hispanic physicians.

## Background

Few studies have reported on the particular perspectives and actual practices of U.S. Hispanic physicians with respect to tobacco counseling and smoking cessation. There is a scarcity of information related to how Hispanic physicians perceive their responsibility to educate patients who smoke. In addition, the literature does not clearly discuss their awareness of available resources for assisting smoking patients, nor how tobacco-use assessment, counseling, and follow-up are incorporated into their actual practice. This is of particular importance considering that Hispanic physicians may confront situations that are particular not only to their patient populations, but also to their own cultural and educational backgrounds. In addition, despite the fact that Hispanic physicians are underrepresented nationally, this group constitutes a considerable professional collective. The American Medical Association's membership includes more than 28,400 Hispanic physicians, [1] and the National Hispanic Medical Association represents more than 36,000 licensed Hispanic physicians. [2] In certain states, Hispanic physicians represent a significant percentage of the total physician population: approximately 11% in Florida, 8% in New Mexico and 7% in Texas [3]. Finally, given that smoking continues to be a priority health behavior problem among Hispanics, and that physicians can potentially play an important role in delivering education messages, [4], assessing the tobacco-related training needs of Hispanic physicians should be considered a priority.

The most recent national data show that current smoking prevalence among Hispanic adults is at 16.7%, [5] and that more than 20% of Hispanic high school students are smokers [6]. This constitutes more than 6 million Hispanic youth and adult smokers in need of assistance. Although the literature indicates that tobacco dependence and desire to quit is prevalent across all racial and ethnic groups, and that smoking cessation interventions have shown to be effective in both the general population as well as minority populations, interventions and treatments must be tailored to the cultural characteristics of the participant population [7,8]. Furthermore, information and education must be communicated in a language that is understood by the smoker [7]. There is a recognized need for programs that are specifically developed to address the health needs of Hispanics, including programs for tobacco use prevention and smoking cessation, [8] and Hispanic physicians could play a key role in attending to these tobacco education and smoking cessation needs. According to the literature, race, culture, and language are important factors among Hispanics when selecting their physicians [9-12]. Therefore, if Hispanics prefer physicians who share their same ethnicity, the Hispanic physician may be most suited to attend to the health education needs of this growing population group.

It is essential to better understand how Hispanic physicians perceive their responsibility about educating patients who smoke, and to shed light on their actual practices related to tobacco use assessment, counseling and follow-up. The purpose of this study was to conduct an assessment of the tobacco intervention practices and training needs of Hispanic physicians through a validated survey instrument.

## Methods

Data was collected through a validated survey developed by the investigators using qualitative and quantitative approaches. The qualitative study involved the analysis of primary data collected through semi-structured taped interviews with nine practicing Hispanic physicians representing various geographic areas in the state of New Mexico. The study protocol was approved by the University of New Mexico Institutional Review Board. Participants were asked to discuss issues related to tobacco use among their patients as well as their personal perspectives and practices regarding tobacco/smoking intervention. Tapes were transcribed and data compiled using a key word coding system that focused on answering the questions of interest for this study.

The results of the qualitative analysis, along with a review of the related literature, informed the development of the initial draft survey, which was then pilot-tested. To assess face and content validity, the survey was presented to a panel of three health education experts who ensured that all domains of interest were appropriately captured, and to a language specialist, who provided feedback on wording and grammar. A second draft was then developed and piloted with thirteen Hispanic physicians with the purpose of improving the face validity of the instrument by involving the participating population. This data also provided information on the administration process. Most participants completed the survey in 10–13 minutes.

Based on the feedback received, a final instrument was developed and psychometric tests conducted. These included test-retest reliability (N = 8) and internal consistency (N = 45). The results of the test-retest Pearson correlation coefficient (see Table 1) indicated acceptable reliability across the eight items used to assess the domain of interest (see Table 3), as typically seen in validation studies with adults [13]. The Cronbach alpha value was 0.81, which is an acceptable level of reliability. Reliability was estimated by computing the average scores across all the items in the domain to produce a scale score. Thus each item is weighted equally in contributing to the total score.

**Table 1:** Test-retest coefficients for each item

Item	Coefficient
1	.98
2	.90
3	.69
4	.81
5	.66
6	.62
7	.82
8	.92

**Table 2:** Characteristics of respondents

Item	n	(%)
Sex		
Female	12	26.7
Male	33	73.3
Age group		
20–35	1	2.2
36–45	22	48.9
46–50	9	20.0
50+	13	28.9
Place of birth		
USA	41	91.1
Mexico	2	4.4
Puerto Rico	2	4.4
Professional category		
Primary care	21	46.7
Specialist	24	53.3
Years of practice in the U.S.		
1–3	3	6.7
3–6	1	2.2
6–10	8	17.8
10+	31	68.9
Type of practice		
Private office	23	51.1
HMO	2	4.4
Hospital	7	15.6
Non-hospital based clinic	4	8.9
Country of medical training		
USA	42	93.3
Mexico	1	2.2
Puerto Rico	1	2.2
Language spoken at home		
English	41	91.1
Spanish	1	2.2
Both	3	6.7
Language most spoken in practice		
English	35	77.8
Spanish	2	4.4
Both	8	17.8

**Table 3:** Physicians' tobacco-related practices in a typical office visit

Intervention	Frequency and percent of physicians who perform the intervention with:				
	
	>80% of patients	61–80% of patients	41–60% of patients	20–40% of patients	<20% of patients
Ask about smoking status	(20) 44.4%	(10) 22.2%	(5) 11.1%	(4) 8.9%	(6) 13.3%
Advise smoking patients to quit	(19) 42.2%	(8) 17.8%	(5) 11.1%	(5) 11.1%	(7) 15.6%
Assist smoking patients	(11) 24.4%	(6) 13.3%	(7) 15.6%	(4) 8.9%	(16) 35.6%
Arrange follow-up for smoking patients	(2) 4.4%	(4) 8.9%	(3) 6.7%	(4) 8.9%	(30) 66.7%
Prescribe NRT or other treatment	(0) 0.0%	(0) 0.0%	(6) 13.3%	(10) 22.2%	(29) 64.4%
Use/refer behavioral change programs	(2) 4.4%	(0) 0.0%	(3) 6.7%	(7) 15.6%	(33) 73.3%
Ask patients about exposure to ETS	(7) 15.6%	(2) 4.4%	(3) 6.7%	(11) 24.4%	(22) 48.9%
Assist patients exposed to ETS	(3) 6.7%	(2) 4.4%	(5) 11.1%	(6) 13.3%	(28) 62.2%

The outcome of the described development process was a 50-item instrument that is population-specific and appropriate for assessing the professional characteristics and tobacco practices and educational needs of Hispanic physicians. In addition to standard demographic questions, the instrument includes items related to country of education and professional training, language spoken at home and in professional practice, and ethnicity and language of the patient population. Items related to physicians' smoking status include past and present use of both cigarettes and cigars (the instrument is available upon request).

Eight items assess physicians' tobacco intervention practices, as follows: 1) ask patients about smoking status, 2) advise smoking patients to quit, 3) assist smoking patients by talking to them about the health risks of smoking, providing materials, or referring, 4) arrange follow-up for smoking patients-follow-up visits or phone calls, 5) prescribe Nicotine Replacement Therapy (NRT) or other cessation treatments to smoking patients, 6) use behavioral change techniques with smoking patients, or refer them to behavioral change programs, 7) ask patients about exposure to secondhand smoke, and 8) assist patients exposed to secondhand smoke in understanding the health risks associated with ETS by talking to them or providing materials. Physicians were asked to indicate the percentage of patients with whom they perform each activity in a typical office visit according to the following scale: <20%, 20–40%, 41–60%, 61–80%, and >80% (see Table 3). Greater than 80% was established as the standard for defining "routine practice." This figure was based on the *Healthy People 2000 Objectives for the Nation*, which established 75% as the benchmark for "routine" tobacco intervention practices. Respondents were also asked to indicate the specific factors which personally prevent them from intervening with patients who smoke, and whether they have access to resources to assist smoking patients. The last section explores tobacco counseling training preferences.

This study used a cross-sectional design that included physicians who were members of the New Mexico Hispanic Medical Society (NMHMS). The NMHMS approved the project and encouraged participation. The unit of analysis and observation for this study consisted of the individual physician. The main selection criterion for inclusion was that participants be practicing physicians. A potential sample population of 81 physicians qualified for the study. A packet including a cover letter, IRB approved informed consent, the survey, and a self-stamped return envelope was mailed to all potential participants. A follow-up letter was sent to non-respondents a month later, and a few weeks later a second packet was mailed to those who had not yet responded. This second packet included a letter offering an incentive to participate (a $15 gift certificate).

## Results

Forty-five surveys, or 55.5% of the initial sample size, were entered into the dataset. All data screening, computation, and analyses were conducted using SPSS 10.0 for Microsoft Windows. Characteristics of respondent physicians are included in Table 2. The majority of respondents were male, in the 36–45 years-of-age group, and born in the U.S. The number of participants who were specialists was slightly higher than that of those who were primary care physicians (Primary care physicians generally include family practice and internal medicine. Pediatricians are sometimes considered primary care physicians for children, adolescents and teenagers while obstetricians/gynecologists are sometimes considered primary care physicians for women. For this study, primary care physicians were considered those who responded "Yes" to the question "Primary Care Physician?"). The majority completed medical training in the U.S., had been practicing for more than 10 years, and indicated that they speak English at home and in their professional practice. Although 26.7% (n = 12) of participant physicians had smoked more than 100 cigarettes in their lifetime, none were current cigarette smokers at the time of completing the survey. Only 4.4% (n = 2) had smoked more than 100 cigars in their lifetime; however, among these, 6.7% (n = 3) reported being current cigar smokers.

Information on the patient population of participant physicians was collected. Overall, respondents estimated that they see about the same percentage of Hispanic/Latino and Anglo/Caucasian patients, 6.7% (n = 3) indicated that their patients speak mostly Spanish, and 22.2% (n = 10) that their patients speak both Spanish and English. Forty percent (n = 18) of participant physicians estimated that about one-fourth of their patient population smoke, 35.6% (n = 16) estimated that about one-fourth suffer from tobacco-related illness, and 28.9% (n = 13) estimated that about half or more suffer from tobacco-related illness.

**Figure 1:**
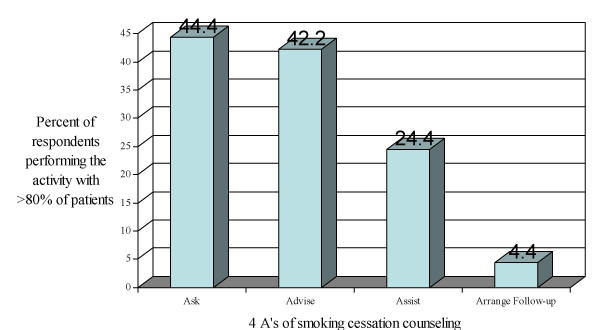
Percentage of physicians performing the 4 A's of smoking cessation Counseling with >80% of patients.

Figure 1 shows the variability in tobacco-related practices among respondents. Less than 45% of participant physicians routinely perform the most basic intervention: asking patients about smoking status and advising smoking patients to quit. Even fewer, 24.4%, routinely assist smoking patients by talking to them about the health risks of smoking, providing education materials or referring them to cessation programs. Only 4.4% routinely arrange follow-up visits or phone calls for smoking patients.

In regard to treating smokers, none of the physicians reported prescribing nicotine replacement therapy or any other type of treatment to more than 60% of smoking patients. To the contrary, the majority (64.4%) indicated that they prescribe cessation treatments to less than 20% of smoking patients. An even smaller percentage, only 4.4%, reported routinely using behavioral change techniques or referring smokers to behavioral change programs. As far as secondhand smoke is concerned, only 15.6% indicated that they routinely ask their patients about exposure to tobacco smoke and 6.7% routinely assist patients exposed to secondhand smoke in understanding the health risks associated with ETS (see Table 3).

Participants were asked to report on the two most frequently encountered barriers preventing them from intervening with patients who smoke by selecting from a list of potential barriers identified during the qualitative study. These barriers were related to available time, reimbursement, training, receptivity of the patient, patient's choice, and language. Of those who reported not always counseling their patients, the most frequently selected barriers included: patients are not receptive (50%, n = 15); counseling takes too much time (33.3%, n = 10); I do not have the proper training to do an intervention (30, n = 9); and counseling time is not reimbursable by third party payers (20%, n = 6). However, 23.3% (n = 7) selected "other" reasons not specified.

Regarding availability and access to tobacco use education and control resources, almost half of respondents (46.6%, n = 21) were not sure about community resources they could use for assisting patients who smoke, and 26.6% (n = 12) indicated that they did not have access to cessation programs or technical support. The majority (73.3%, n = 33) responded either that they were not sure whether they had the materials they needed in their office to adequately educate smokers, or indicated they simply did not have the necessary materials. When asked about resources they would use should they become available, the majority indicated that they would use bilingual Spanish materials, including cessation programs (60.0%, n = 27), education materials (62.2%, n = 28), and quitting self-help guides (64.4%, n = 29).

With respect to tobacco-related training, almost all participants (97.7%, n = 44) indicated a preference for educating themselves about tobacco intervention skills through articles in scientific/professional journals. Written materials, such as manuals, brochures, etc., were the second preferred choice selected by 60% (n = 27) of respondents, while 35.5% (n = 16) selected continuing education courses.

Factorial analyses of variance were conducted to determine whether there were significant mean score differences on the variable of interest by gender, by type of physician (primary care or not), or by the interaction of gender and type of physician. Results indicated a main effect of gender on tobacco intervention practice, with male physicians scoring significantly more positively than female physicians. Male physicians (M = 20.20, SD = 6.50) scored nearly 30% higher than female physicians (M = 14.75, SD = 6.14) (see Table 4). There was no significant main effect related to type of physician, nor was there an interaction effect (gender by type of physician) on tobacco-related practices.

**Table 4:** Factorial ANOVA results indicating main and interaction effects of gender and type of physician on tobacco-related practices

Source of Variance	Sum of Squares	df	Mean Square	F	p
Gender	287.89	1	287.89	6.87	.01*
Type of Physician	37.38	1	37.38	.89	.35
Gender by Type of Physician	32.24	1	32.24	.77	.39
Error	1717.12	41	41.88		

*R Squared = .152

## Discussion

The study provides relevant demographic information related to respondents' smoking status, ethnicity, and language use. Physicians who participated in the study reported both low prevalence of cigarette smoking and low levels of smoking intervention. It should be noted some studies have shown no relation between a physician's own smoking status and tobacco-related practices [14,15]. Regarding the smoking status of the patient population, it is interesting that a high percentage of respondents estimated that about 25% of the patients they see are smokers, a figure which is consistent with the national average. It is even more significant that nearly 30% of respondents estimated that half of their patients suffer from tobacco-related illnesses. This points to the enormous impact of tobacco on health care costs, and suggests that costs could be significantly reduced by eliminating smoking among the study population.

It is important to mention the demographic information related to ethnicity that emerged from the data. Respondents estimated that 47% of their patients are Hispanic, which is higher than the estimated Hispanic population of New Mexico at the time of the study (42%) [16]. Although this figure was estimated by respondents, it would be worth exploring further because it may support the hypothesis that Hispanics prefer physicians who are of their own race/ethnicity. If this is true, the Hispanic physician may be well posited for educating the Hispanic population and delivering tobacco education and smoking cessation interventions to this group.

Results related to tobacco intervention were diverse. Although the qualitative study indicated that participants understand the health burden caused by tobacco use, and feel responsible for intervening with patients who smoke, their actual practices do not reach a level that would significantly contribute to tobacco control. It is significant that less than half of the respondents in this study routinely ask about smoking status, particularly because tobacco use is generally recorded in the medical chart. This was confirmed by the interviews during the qualitative study. Respondents indicated that tobacco use is assessed during intake, and that they know whether a patient uses tobacco by looking at the chart. This may be relevant when taking a systems approach to smoking cessation: simply institutionalizing the recording of smoking status in the medical chart may have a very limited impact on cessation intervention by physicians. Similar conclusions have been reported by Zapka et al [17].

In this study, less than half of respondents reported routinely asking patients about smoking status and advising smoking patients, and less than one fourth routinely talk with patients about the health risks of smoking, provide education materials, or refer them to cessation programs. These results compare negatively with data from national studies, and suggest that the practices of Hispanic physicians may negatively compare with those of their peers. Goldstein et al [15]. assessed community-based primary care physicians and found that 67% "ask" about smoking status during more than 80% of all patient visits; 74% "advise" smoking patients to quit; 35% "assist" smoking patients by talking to them, providing materials, prescribing gum or referring; and 8% "arrange" follow-up visits or phone calls. Similarly, the 2004 State of Health Care Quality report found that more than 68% of current smokers were advised to quit by their practitioners [18].

Whether the lower levels of intervention revealed in this study confirm a national pattern among Hispanic physicians is not clear, given that few national studies have specifically identified Hispanic physicians' level of smoking intervention. However, some studies have found differences in smoking intervention between Hispanic physicians and physicians from other ethnic/racial groups. A study with prenatal, pediatric and WIC providers in greater Boston, Mass., found lower smoking intervention performance among Hispanic providers in comparison with Non-Hispanic Black and White providers, although differences were not significant when controlling for other variables [17].

Regarding ETS, few participants in this study routinely address secondhand smoke in their daily practice. The literature does not provide much information on ETS assessment and counseling by Hispanic physicians, but it is important to note that they may be in a unique position to address the problem. Strong family ties constitute a traditional Hispanic value, and smoking patients may be more willing to follow the advice of a physician not to smoke at home or around family members when it is presented within the context of *familismo *or family.

The principal barriers to smoking cessation intervention identified in this study include: perceived lack of receptivity by patients, time, lack of training, and lack of economic reimbursement for counseling. These barriers are similar to those found in other studies [17,19,20]. These results indicate that both systematic and individual approaches need to be incorporated into training programs.

Interventions should also consider whether physicians have access to resources for assisting smoking patients. In this study, almost half of the participants indicated that they were not sure about the availability of community resources for smoking cessation, one fourth felt they did not have access to programs and support, and three-fourths were simply not sure or did not have in-office access to all of the materials they needed for educating smokers. Most participants indicated the need for bilingual materials and cessation programs. The qualitative study supported these results. None of the physicians who were interviewed knew about the state tobacco control program in NM.

Finally, almost all respondents indicated a preference for educating themselves about interventions and skills through articles published in scientific/professional journals. This is a significant finding, as most health professionals subscribe to scientific journals. While research papers do not generally discuss the practical applicability of the results, journals interested in contributing to educating professionals on tobacco intervention should require authors to discuss the link between research and practice. The second preferred choice was "other written materials" (brochures, pamphlets, etc.). Only 37.7% (n = 17) selected continuing education courses.

## Limitations

A number of limitations of this study should be mentioned. First, the sample size was small, although it represents approximately 15% of the Hispanic physician population in NM [3]. Additionally, all participants were members of a professional organization, which may have influenced their responses. In addition, survey data were self-reported, and, although the survey instrument demonstrated good validity and reliability, providers' self-reported practices may be less valid than data obtained from other sources [23]. Additionally, self-reported practices may be more positive among respondents than among non-respondents. Finally, due to the small sample size of the study, results must be interpreted cautiously when generalizing to the general Hispanic physician population. Considering these limitations, the implementation of a follow-up national study with a larger sample and a more diverse participant population is recommended. It would be useful to compare the perceptions, opinions, and practices of Hispanic physicians of diverse nationalities and educational backgrounds. A larger and more comprehensive study would provide valuable information that could potentially be used to inform the development of interventions to educate Hispanic physicians about tobacco assessment, counseling, and follow-up.

## Conclusion

To the knowledge of these investigators, this is the most comprehensive study that has explored the tobacco intervention practices of U.S. Hispanic physicians. In addition to the results of the qualitative and quantitative studies, another positive outcome of this investigation was the development of a survey instrument that showed good validity and reliability. This instrument should prove to be of interest to federal health agencies and managed care organizations, for investigating Hispanic physicians' tobacco intervention practices. According to the literature, standard procedures for collecting data on physicians' tobacco education and smoking cessation practices present several methodological challenges. These include the use of survey instruments that have not undergone the necessary processes to demonstrate their validity and reliability. The use of such instruments may compromise the internal validity and results of an investigation [21,22]. Furthermore, the instrument developed for this study includes questions not generally found in other surveys which make the instrument more appropriate for assessing the characteristics of Hispanic physicians, including items related to level of acculturation, language proficiency and training needs.

In summary, although the literature has consistently reported on physicians' low level of compliance with smoking cessation guidelines and recommendations, this study suggests that Hispanic physicians may be in greater need of training and resources than other groups. The results of this study compare negatively with data from national studies on physicians in general. Regarding "best practices" for delivering training initiatives to Hispanic physicians, the results of this study point to self-education through professional/scientific journals. However, this result must be further investigated. It could be that Hispanic physicians prefer to learn about tobacco intervention by reading scientific journals, but it may turn out that this approach is not conducive to increased tobacco intervention. Connecting research and practice is an issue of concern in health education, and journal articles will certainly not provide adequate training if researchers do not explain the practical applicability of their research findings.

Given the growth of the Hispanic population in the U.S., and the demonstrated potential role of physicians in smoking cessation, more resources should be dedicated to identifying the tobacco intervention needs of Hispanic physicians, and to improving their cessation practices.

## Competing interests

The author(s) declare that they have no competing interests.

## Authors' contributions

FGSM designed the study, conducted data collection and analysis, and wrote the first draft of the manuscript. RLP contributed to study design, data analysis, interpretation of results, and technical and editorial review. HEJ contributed to study design and editorial review. CEH contributed to data analysis and manuscript development, including technical and editorial review. XU-R contributed to interpretation of results and technical and editorial review. WMK contributed to study design, interpretation of results and technical review.

## Pre-publication history

The pre-publication history for this paper can be accessed here:



## References

[B1] Total physicians by race/ethnicity-2003.

[B2] National Hispanic Medical Association. http://www.nhmamd.org/about.htm.

[B3] Distribution of nonfederal physicians by race, 2003. http://statehealthfacts.org.

[B4] Soto Mas F, Papenfuss R (1997). Medicina y salud pública. Médico Interamericano.

[B5] Adult cigarette smoking in the United States: current estimates. May, 2004. http://www.cdc.gov/tobacco/factsheets/AdultCigaretteSmoking_FactSheet.htm.

[B6] Youth and tobacco use: current estimates. December, 2003. http://www.cdc.gov/tobacco/research_data/youth/Youth_Factsheet.htm.

[B7] DHHS (2000). Treating tobacco use and dependence. Clinical Practice Guideline.

[B8] DHHS (2000). Healthy People 2010: understanding and improving health.

[B9] Saha S, Taggart SH, Komaromy M, Bindman AB (2000). Do patients choose physicians of their own race?. Health Aff.

[B10] Cooper-Patrick L, Gallo JJ, Gonzales JJ, Vu HT, Powe NR, Nelson C, Ford D (1999). Race, gender, and partnership in the patient-physician relationship. JAMA.

[B11] Morales L, Cunningham W, Brown J, Hays R (1999). Are Latinos less satisfied with communication by health care providers?. J Gen Intern Med.

[B12] David R, Rhee M (1998). The impact of language as a barrier to effective health care in an underserved urban Hispanic community. Mt Sinai J Med.

[B13] Willet W (1990). Nutritional epidemiology.

[B14] Saywell RM, Jay SJ, Lukas PJ, Casebeer LL, Mybeck KC, Parchman ML, Haley AJ (1996). Indiana family physicians attitudes and practices concerning smoking cessation. Indiana Medicine.

[B15] Goldstein MG, DePue JD, Monroe AD, Lessne CW, Rakowski W, Prokhorov A, Niaura R, Dube CE (1998). A population-based survey of physician smoking cessation counseling practices. Prev Med.

[B16] New Mexico QuickFacts. http://quickfacts.census.gov/qfd/states/35000.html.

[B17] Zapka JG, Pbert L, Stoddard AM, Ockene JK, Valentine Goins K, Bonollo D (2000). Smoking cessation counseling with pregnant and postpartum women: a survey of community health centers. Am J Public Health.

[B18] The State of Health Care Quality 2004. NCQA\Publications\SOHC 2004. http://www.ncqa.org/communications/sohc2004/ma_smoking.htm.

[B19] Cabana MD, Rand CS, Powe NR, Wu AW, Wilson MH, Abboud PA, Rubin HR (1999). Why don't physicians follow clinical practice guidelines?. JAMA.

[B20] Jaen C, Crabtree B, Zyzansky S, Goodwin M, Stange K (1998). Making time for smoking cessation counseling. J Fam Pract.

[B21] Crooks C, Kenney E, Elder J, Levitz M, Johnson M, Bal D (1993). Tobacco Control activities of primary care physicians in California. Eval Health Prof.

[B22] Zapka JG, Fletcher KE, Ma Y, Pbert L (1997). Physicians and smoking cessation: development of survey measures. Eval Health Prof.

[B23] Montano DE, Phillips WR (1995). Cancer screening by primary care physicians: a comparison of rates obtained from physician self-report, patient survey, and chart audit. Am J Public Health.

